# Summary of the European Directive 2013/59/Euratom: essentials for health professionals in radiology

**DOI:** 10.1007/s13244-015-0410-4

**Published:** 2015-05-27

**Authors:** European Society of Radiology (ESR)

**Affiliations:** Neutorgasse 9/2, 1010 Vienna, Austria

**Keywords:** Protection, Radiation, Legislation, Medical, European Union, Government regulations

## Abstract

The aspects of the new European Directive 2013/59/Euratom most relevant to diagnostic imaging and intervention are summarised. The Directive, laying down basic safety standards for protection against the dangers from exposure to ionising radiation, emphasises the need for justification of medical exposure (including asymptomatic individuals), introduces requirements concerning patient information and strengthens those for recording and reporting doses from radiological procedures, the use of diagnostic reference levels, the availability of dose-indicating devices and the improved role and support of the Medical Physics Experts in imaging. Relevant changes include new definitions, a new dose limit for the eye lens, non-medical imaging exposures, procedures in asymptomatic individuals, the use and regular review of diagnostic reference levels (including interventional procedures), dosimetric information in imaging systems and its transfer to the examination report, new requirements on responsibilities, the registry and analysis of accidental or unintended exposure and population dose evaluation (based on age and gender distribution). These changes will require Member States, the radiology community and the industry to adapt regulations, practices and equipment for a high standard of radiation safety. By 6 February 2018, the Directive has to be transposed into the national legislation of the Member States of the European Union.

*Main messages*

• *The new European Basic Safety Standards Directive impacts radiology departments*

• *Changes in justification, patient information, responsibilities and dose reporting are most significant*

• *Diagnostic reference levels and the role of medical physics experts are clarified*

• *Dose limits to the eye lens are lower than in the previous directive*

• *Responsibilities in radiation safety have been defined*

## Introduction

The new European Directive 2013/59/Euratom [1], laying down basic safety standards (BSS) for protection against the dangers arising from exposure to ionising radiation and repealing Directives 89/618/Euratom, 90/641/Euratom, 96/29/Euratom, 97/43/Euratom and 2003/122/Euratom, is expected to have a relevant and positive impact on European radiology.

The basic safety standards take into account the new recommendations of the International Commission on Radiological Protection (ICRP) [2, 3] and are revised in the light of new scientific evidence and operational experience.

The Directive was unanimously adopted by the Council of the European Union (EU) on 5 December 2013 after 4 years of work by different European scientific and technical committees.^1^ The press release after the Council meeting held in Brussels on 5 December 2013 highlighted that the new Directive, under which the Member States will establish legal requirements and an appropriate regime of regulatory control, reflects a system of radiation protection based on the principles of justification, optimisation and dose limitation for all exposure situations. Dose limits shall not apply to medical exposures.

According to the new Directive, a high level of competence and a clear definition of responsibilities and tasks among all professionals involved in medical exposure are fundamental to ensure adequate protection of patients undergoing medical radiodiagnostic and radiotherapeutic procedures. This applies to medical doctors, dentists and other health professionals entitled to take clinical responsibility for individual medical exposures, to medical physics experts and to other professionals carrying out practical aspects of medical radiological procedures, such as radiographers and technicians in radiodiagnostic medicine, nuclear medicine and radiotherapy.

Furthermore, the Directive provides radiation protection education, training and provision of information. The Member States will have 4 years to transpose this Directive into national legislation.

The most relevant changes in the new Directive in comparison to the existing ones—96/29/Euratom [4] on the protection of workers and the general public and 97/43/Euratom [5] on medical exposures—are summarised in Tables 1 and 2.

**Table 1:** Most relevant changes for radiology imaging in Directive 2013/59/Euratom

1. New set of definitions
2. New dose limit for the lens of the eyes
3. Consideration of occupational doses in justification and optimisation
4. Regulation for radiological procedures in asymptomatic individuals
5. Use and regular review of diagnostic reference levels (including interventional)
6. Education and training (out of the chapter on medical exposures)
7. Responsibilities (the full Art. 57 should be analysed)
8. Role of medical physics experts in diagnostic and interventional procedures
9. New requirements for equipment in use
10. Procedures: optimisation process, clinical protocols and clinical audit
11. Registry and analysis of accidental or unintended exposures
12. Population dose evaluation taking into account age distribution and gender
13. Old “medico-legal” and new “non-medical imaging exposures”

Chapter VII in the new Directive deals with medical exposures but other chapters contain articles relevant to radiology imaging (see Table 2).

**Table 2:** Other requirements of Directive 2013/59/Euratom with high relevance to imaging

Dose constraints for occupational, public and medical exposure (Art. 6)
Dose limits for occupational exposure including the new limit for the lens of the eyes of 20 mSv in a single year (Art. 9)
Pregnant worker protection (Art. 10)
Education, information and training in the field of medical exposure (Art. 18)
Chapter VI on occupational exposures, in particular:
• Operational protection of exposed workers (Art. 32)
• Operational protection of apprentices and students (Art. 33)
• Consultations with radiation protection experts (Art. 34)
• Controlled and supervised areas (Arts. 37-38)
• Radiological surveillance of the workplace (Art. 39)
• Categorisation of exposed workers (Art. 40)
• Individual monitoring and access to the results (Arts. 41 and 44)
• Medical surveillance of exposed workers (Art. 45)

The Directive distinguishes between existing, planned and emergency exposure situations. Taking into account this new framework, the Directive covers all exposure situations and all categories of exposure, namely occupational, public and medical.

The new Directive provides minimum rules, and Member States should be free to adopt or maintain more stringent measures in the subject matter covered by the Directive, without prejudice to the free movement of goods and services in the internal European Union market.

## New set of definitions

The definition of “medical exposure” in the new Directive is similar to the previous one, but excludes the “medico-legal”, now called “non-medical imaging exposure”. The relevant definitions (with some changes in comparison to the previous Directives) are included in the Annex.

## Occupational dose limits and new limit for the lens of the eye

New scientific information on tissue reactions calls for better protection of the eye lens and, following the ICRP guidance, the new Directive modifies the occupational dose limit for the eye lens to 20 mSv/year from the previous value of 150 mSv/year.

Articles 9 and 11 on dose limits for occupational exposure and dose limits for apprentices and students indicate that:

- Dose limits for occupational exposure apply to the sum of annual occupational exposures of a worker from all authorised practices.
- The limit on the effective dose shall be 20 mSv in any single year (in special circumstances 50 mSv if the average annual dose over any 5 consecutive years does not exceed 20 mSv).
- The limit on the equivalent dose for the lens of the eye shall be 20 mSv in a single year or 100 mSv in any 5 consecutive years subject to a maximum dose of 50 mSv in a single year.
- The limit on the equivalent dose for the skin and extremities shall be 500 mSv in a year.
- Apprentices and students (aged 16–18 years) have more restrictive dose limits: an effective dose of 6 mSv in a year and equivalent doses of 15 mSv in a year to the lens of the eye as well as of 150 mSv in a year to the skin and the extremities.
- In addition, Art. 40 establishes the categorisation of exposed workers. Workers are required to be classified as ‘category A’ (i.e., subject to individual monitoring and medical surveillance) if (a) an effective dose greater than 6 mSv per year or (b) an equivalent dose greater than 15 mSv per year for the lens of the eye or (c) greater than 150 mSv per year for the skin and extremities might be expected.

Article 10 deals with pregnant and breastfeeding workers:

- As soon as a pregnant worker informs the employer of the pregnancy the employment conditions shall assure that the equivalent dose to the unborn child is as low as reasonably achievable and unlikely to exceed 1 mSv during at least the remainder of the pregnancy.
- As soon as workers inform the employer that they are breastfeeding an infant, they shall not be employed in work involving a significant risk of intake of radionuclides or of bodily contamination.

## Consideration of occupational doses in justification and optimisation

Articles 55 and 56 in Chap. VII on Medical Exposures maintain most of the requirements of the previous 97/43/Euratom Directive, but the explicit consideration of occupational doses in justification and optimisation (as made by ICRP) in some previous articles should be noted.

Article 19.4. Practices involving medical exposure shall be justified both as a class or type of practice, taking into account medical and, where relevant, associated occupational and public exposures, and at the level of each individual medical exposure.

Article 32.b. Member States shall ensure that the operational protection of exposed workers is based on optimisation of radiation protection in all working conditions, including occupational exposures as a consequence of practices involving medical exposures.

## Regulation for radiological procedures in asymptomatic individuals

A new article (55.2.h) on justification has been added, concerning medical radiological procedures on asymptomatic individuals, to be performed for early disease detection. Such procedures should either be part of a health screening programme or require specific documented justification for that individual by the practitioner, in consultation with the referrer, following guidelines from relevant medical scientific societies and the competent authority. Special attention shall be given to the provision of information to the individual subject to medical exposure.

## Use and regular review of diagnostic reference levels (including interventional)

The new Directive strengthens and expands the previous requirements regarding diagnostic reference levels.

Article 56.2. Member States shall ensure the establishment, regular review and use of diagnostic reference levels for radiodiagnostic examinations, having regard to the recommended European diagnostic reference levels where available, and where appropriate, for interventional radiology procedures and the availability of guidance for this purpose.

Article 58.f. underlines the need for appropriate local reviews whenever diagnostic reference levels are consistently exceeded and requires that the corresponding corrective action is taken without undue delay.

## Education, information and training in the field of medical exposure

Article 18 of the new Directive deals with education, information and training in the field of medical exposure. This article is now part of Chap. VII (Medical Exposures) and its content is the same as in the previous 97/43/Euratom Directive. Article 59 on “training and recognition” in the new Directive refers to this Art. 18.

- Member States shall ensure that practitioners and the individuals involved in the practical aspects of medical radiological procedures have adequate education, information and theoretical and practical training for the purpose of medical radiological practices, as well as relevant competence in radiation protection.
- For this purpose Member States shall ensure that appropriate curricula are established and shall recognise the corresponding diplomas, certificates or formal qualifications.
- Individuals undergoing relevant training programmes may participate in practical aspects of medical radiological procedures.
- Member States shall ensure that continuing education and training after qualification are provided and, in the special case of the clinical use of new techniques, training is provided on these techniques and the relevant radiation protection requirements.
- Member States shall encourage the introduction of a course on radiation protection in the basic curriculum of medical and dental schools.

## Responsibilities

Article 57 deals with responsibilities and contains new requirements regarding the optimisation process and the provision of information to patients.

1. Member States shall ensure that:
  - Any medical exposure takes place under the clinical responsibility of a practitioner;
  - The practitioner, the medical physics expert and those entitled to carry out practical aspects of medical radiological procedures are involved, as specified by Member States, in the optimisation process;
  - The referrer and the practitioner are involved, as specified by Member States, in the justification process of individual medical exposures;
  - Wherever practicable, prior to the exposure taking place, the practitioner or the referrer, as specified by Member States, ensures that the patient or his/her representative is provided with adequate information relating to the benefits and risks associated with the radiation dose from the medical exposure. Similar information as well as relevant guidance shall be given to carers and comforters, in accordance with point (b) of Art. 56(5).
2. Practical aspects of medical radiological procedures may be delegated by the undertaking or the practitioner, as appropriate, to one or more individuals entitled to act in this respect in a recognised field of specialisation.

## Role of the medical physics expert in imaging

Article 58 (procedures), part d, indicates that a medical physics expert shall be involved in radiodiagnostic and interventional radiology practices involving high doses as referred to in point (c) of Art. 61(1) (i.e. interventional radiology and computed tomography). For other medical radiological practices, a medical physics expert shall be involved as appropriate, depending on the radiological risk posed by the practice, for consultation and advice.

Article 83 defines the responsibilities of the medical physics expert:

Member States shall require the medical physics expert to act or give specialist advice as appropriate on matters relating to radiation physics for implementing the requirements set out in Chap. VII and in point (c) of Art. 22(4) of the Directive (i.e. “Practices involving the deliberate exposure of humans for non-medical imaging purposes”).

Member States shall ensure that depending on the medical radiological practice, the medical physics expert takes responsibility for dosimetry, including physical measurements for evaluation of the dose delivered to the patient and other individuals subject to medical exposure, give advice on medical radiological equipment and contribute in particular to the following:

- Optimisation of the radiation protection of patients and other individuals subject to medical exposure, including the application and use of diagnostic reference levels;
- The definition and performance of quality assurance of the medical radiological equipment;
- Acceptance testing of medical radiological equipment;
- The preparation of technical specifications for medical radiological equipment and installation design;
- The surveillance of the medical radiological installations;
- The analysis of events involving, or potentially involving, accidental or unintended medical exposures;
- The selection of equipment required to perform radiation protection measurements;
- The training of practitioners and other staff in relevant aspects of radiation protection;

It is also required that the medical physics expert shall, where appropriate, liaise with the radiation protection expert.

## New requirements for equipment in use

Article 60.3 (equipment) underlines the responsibility of Member States to ensure that:

- The use of fluoroscopy equipment without a device to automatically control the dose rate, or without an image intensifier or equivalent device, is prohibited.
- Any equipment used for interventional radiology has a device or a feature informing the practitioner and those carrying out practical aspects of the medical procedures of the quantity of radiation produced by the equipment during the procedure. Equipment installed prior to 6 February 2018 may be exempted from this requirement.
- Any equipment used for interventional radiology and computed tomography and any new equipment used for planning, guiding and verification purposes have a device or a feature informing the practitioner, at the end of the procedure, of relevant parameters for assessing the patient dose.
- Equipment used for interventional radiology and computed tomography has the capacity to transfer the information required under 3(d) to the record of the examination. Equipment installed prior to 6 February 2018 may be exempted from this requirement.
- Without prejudice to points (c), (d) and (e) of paragraph 3, new medical radiodiagnostic equipment producing ionising radiation has a device, or an equivalent means, informing the practitioner of relevant parameters for assessing the patient dose. Where appropriate, the equipment shall have the capacity to transfer this information to the record of the examination.

## Procedures

Article 58.a now requires the establishment of written protocols for every type of standard medical radiological procedure and for each piece of equipment, but also “for relevant categories of patients”.

Article 58.b (procedures) indicates that Member States shall ensure that “Information relating to patient exposure forms part of the report of the medical radiological procedure”.

In Art. 58.c the previous term “recommendations concerning referral criteria” has been replaced with “referral guidelines for medical imaging” to better reflect the European practice.

Article 58.e maintains the requirements on clinical audits as in the previous Directive 97/43/Euratom [5], stating that “clinical audits are carried out in accordance with national procedures”.

## Registry and analysis of the accidental or unintended irradiations

Article 63 introduces a new set of requirements for registration and analysis of accidental and unintended medical exposures.

Member states shall ensure that:

(c) For all medical exposures the undertaking implements an appropriate system for the record keeping and analysis of events involving or potentially involving accidental or unintended medical exposures, commensurate with the radiological risk posed by the practice;
(d) Arrangements are made to inform the referrer and the practitioner, and the patient or their representative, about clinically significant unintended or accidental exposures and the results of the analysis;
(e) (i) The undertaking declares as soon as possible to the competent authority the occurrence of significant events as defined by the competent authority;
  (ii) The results of the investigation and the corrective measures to avoid such events are reported to the competent authority within the time period specified by the Member State;
(f) Mechanisms are in place for the timely dissemination of information relevant to radiation protection in medical exposure regarding lessons learned from significant events.

## Population dose evaluation taking into account the age distribution and the gender

Article 64 states that Member States shall ensure that the distribution of individual dose estimates from medical exposure for radiodiagnostic and interventional radiology purposes are determined, taking into consideration where appropriate the distribution by age and gender of the exposed.

## Old medico-legal and new non-medical imaging exposures

The “medico-legal” exposures introduced in Directive 97/43/Euratom have been replaced in the new Directive by the “non-medical imaging exposures” defined as deliberate exposure of humans for imaging purposes where the primary intention of the exposure is not to bring a health benefit to the individual being exposed. The Directive adopts a different approach for procedures using medical radiological equipment and for procedures not using such equipment.

The practices using medical radiological equipment, as defined in Annex V, include:

1. Radiological health assessment for employment purposes;
2. Radiological health assessment for immigration purposes;
3. Radiological health assessment for insurance purposes;
4. Radiological evaluation of the physical development of children and adolescents with a view to a career in sports, dancing, etc.
5. Radiological age assessment;
6. Use of ionising radiation for the identification of objects concealed within the human body.

The Directive requires practices using medical radiological equipment to be placed under appropriate regulatory control and to be justified at three levels: before being generally adopted, for each particular application and for the particular exposed individual. The Directive also requires reviews of the general and particular justification of such practices.

Article 22.3 indicates that Member States may exempt justified practices involving non-medical imaging exposure using medical radiological equipment from the requirement for dose constraints according to point (b) of Art. 6(1) (i.e. dose constraint shall be set for the individual dose that members of the public receive from the planned operation of a radiation source) and from the dose limits set out in Art. 12 (i.e. dose limits for public exposure).

Article 22.4.c requires that for procedures using medical radiological equipment, the relevant requirements identified for medical exposure as set out in Chap. VII (Medical Exposures) are applied, including those for equipment, optimisation, responsibilities, training and special protection during pregnancy and the appropriate involvement of the medical physics expert.

## Conclusion

The changes introduced with Council Directive 2013/59/Euratom will require Member States, the radiology community (including medical physics experts) and the industry to adapt their regulations, procedures and equipment to the new high standards of radiation safety.

Member States will have 4 years for the transposition of the Directive into national legislation (by 6 February 2018).

## Appendix: Relevant definitions

Accidental exposure: An exposure of individuals, other than emergency workers, as a result of an accident.
Carers and comforters: Individuals knowingly and willingly incurring an exposure to ionising radiation by helping, other than as part of their occupation, in the support and comfort of individuals undergoing or having undergone medical exposure.
Clinical audit: A systematic examination or review of medical radiological procedures that seeks to improve the quality and outcome of patient care through structured review, whereby medical radiological practices, procedures and results are examined against agreed standards for good medical radiological procedures, with modification of practices, where appropriate, and the application of new standards if necessary.
Clinical responsibility: Responsibility of a practitioner for individual medical exposures, in particular, justification; optimisation; clinical evaluation of the outcome; cooperation with other specialists and staff, as appropriate, regarding practical aspects of medical radiological procedures; obtaining information, if appropriate, on previous examinations; providing existing medical radiological information and/or records to other practitioners and/or the referrer, as required; and giving information on the risk of ionising radiation to patients and other individuals involved, as appropriate.
Diagnostic reference levels: Dose levels in medical radiodiagnostic or interventional radiology practices, or, in the case of radio-pharmaceuticals, levels of activity, for typical examinations for groups of standard-sized patients or standard phantoms for broadly defined types of equipment.
Dose constraint: A constraint set as a prospective upper bound of individual doses, used to define the range of options considered in the process of optimisation for a given radiation source in a planned exposure situation.
Interventional radiology: The use of X-ray imaging techniques to facilitate the introduction and guidance of devices in the body for diagnostic or treatment purposes.
Medical exposure: Exposure incurred by patients or asymptomatic individuals as part of their own medical or dental diagnosis or treatment, and intended to benefit their health, as well as exposure incurred by carers and comforters and by volunteers in medical or biomedical research.
Medical physics expert: An individual or, if provided for in national legislation, a group of individuals, having the knowledge, training and experience to act or give advice on matters relating to radiation physics applied to medical exposure, whose competence in this respect is recognised by the competent authority.
Medical radiological: Pertaining to radiodiagnostic and radiotherapeutic procedures and interventional radiology or other medical uses of ionising radiation for planning, guiding and verification purposes.
Medical radiological installation: A facility where medical radiological procedures are performed.
Medical radiological procedure: Any procedure giving rise to medical exposure.
Non-medical imaging exposure: Any deliberate exposure of humans for imaging purposes where the primary intention of the exposure is not to bring a health benefit to the individual being exposed.
Practitioner: A medical doctor, dentist or other health professional who is entitled to take clinical responsibility for an individual medical exposure in accordance with national requirements.
Radiodiagnostic: Pertaining to in-vivo diagnostic nuclear medicine, medical diagnostic radiology using ionising radiation and dental radiology.
Radiotherapeutic: Pertaining to radiotherapy, including nuclear medicine for therapeutic purposes.
Referrer: A medical doctor, dentist or other health professional who is entitled to refer individuals for medical radiological procedures to a practitioner, in accordance with national requirements
Unintended exposure: Medical exposure that is significantly different from the medical exposure intended for a given purpose.

## Footnotes

1 The Council of the EU is the institution representing the member states’ governments. Also informally known as the EU Council, it is where national ministers from each EU country meet to adopt laws and coordinate policies.
